# The ESA Swarm mission to help ionospheric modeling: a new NeQuick topside formulation for mid-latitude regions

**DOI:** 10.1038/s41598-019-48440-6

**Published:** 2019-08-22

**Authors:** M. Pezzopane, A. Pignalberi

**Affiliations:** Istituto Nazionale di Geofisica e Vulcanologia, Via di Vigna Murata 605, 00143 Rome, Italy

**Keywords:** Space physics, Atmospheric dynamics

## Abstract

The ionospheric topside representation made by the NeQuick model is improved by correcting the *H*_0_ parameter used by the model to calculate the topside scale height. The task is accomplished by fitting the NeQuick topside analytical function through two anchor points: the F2-layer absolute electron density maximum; the electron density value as measured by Swarm satellites from December 2013 to September 2018. Specifically, two-dimensional grids of *H*_0_ as a function of *fo*F2 and *hm*F2 are obtained, one by employing data from Swarm A and Swarm C, both flying at about 460 km of altitude, and the other one by employing data from Swarm B, flying at about 520 km of altitude. These two grids are used to calculate corrected values of *H*_0_, which allows a more reliable description of the topside region. The new NeQuick formulation is statistically validated by comparing corresponding modeled vertical total electron content values to those derived from COSMIC/FORMOSAT-3 measured Radio Occultation profiles, and those measured by Swarm satellites. The results show that the proposed formulation significantly improves the topside description made by the NeQuick model at mid latitudes, for both high and low solar activities. The International Reference Ionosphere model might benefit of its inclusion.

## Introduction

The topside part of the ionosphere extends from the F2-layer absolute maximum of the electron density to the plasmasphere^1^. The topside ionosphere is characterized by a decreasing of the electron density mainly due to the smooth transition from an atmosphere dominated by heavy O^+^ ions, in the F region, to less heavy H^+^ ions above on (to be precise, for a more accurate description also the dependence on the plasma temperature should be considered). This behavior is described by means of monotonically decreasing analytical functions dependent on a parameter called plasma scale height^2^.

An exact description of the plasma scale height requires the knowledge of the physical state, in terms of temperature, and of the chemical state, in terms of mean ion mass, of the plasma for the whole topside profile. However, such accurate knowledge is not currently available with the required spatial and temporal coverage. A simpler and more practical approach relies on the use of measured topside electron density values, which allows the calculation of effective scale height values (e.g.^3,4^). Indeed, the effective scale height is an empirical parameter calculated by fitting measured electron density values with analytical functions, in order to obtain the most reliable representation of the topside vertical electron density distribution. Over the years, different techniques have been employed by many authors to model the effective scale height^5^.

A reliable modeling of the topside ionosphere, hence of the corresponding effective scale height, is one of the most difficult task when modeling the vertical electron density profile of the ionosphere. For example, the International Reference Ionosphere (IRI) model^6^ is not able to represent properly the real features of the topside ionosphere^7–9^. One of the topside options proposed by the IRI model is the NeQuick formulation^10–12^, which often proved to be the most reliable^9^, based on a semi-Epstein layer with an empirically formulated scale height varying with altitude. The NeQuick’s topside scale height assumes the value *H*_0_ at the height *hm*F2 of the absolute maximum of the electron density *Nm*F2 and then increases with altitude (see Eq. (2.3)).

In the present work, on the base of both the F2-layer peak values (*hm*F2 and *Nm*F2) provided by the International Reference Ionosphere UPdate (IRI UP) method^13^ and the electron density values provided by Langmuir probes on-board Swarm satellites, and according to the procedure proposed by Pignalberi, *et al*.^5^, a new formulation of the *H*_0_ parameter is proposed. A statistical validation of the new *H*_0_ formulation shows that it significantly improves the topside description made by the NeQuick model at mid latitudes, for both high and low solar activities.

Swarm Langmuir probes and the IRI UP method, on whose data is based the new proposed formulation of the NeQuick’s *H*_0_ parameter, are briefly described in the “Data and method” section. The same section describes also in detail the several steps followed to output the new formulation of *H*_0_, and the COSMIC and Swarm vertical total electron content (vTEC) datasets used for its statistical validation, which will be the subject of the “Results and validation” section. Conclusions are the subject of last section.

## Data and Method

By forcing the NeQuick topside analytical formulation to join the F2-layer peak characteristics and an anchor point in the topside profile, a corrected value for the *H*_0_ parameter can be inferred by minimization. In this work, the F2-layer characteristics are provided by the IRI UP method^13,14^, while the altitude and the electron density of the above anchor point are provided by Langmuir probes on-board Swarm satellites.

### IRI UP F2-layer characteristics calculation and ionosonde data

IRI UP^13,14^ is an empirical data-assimilation method able to update the F2-layer description made by the IRI model^6^ through the assimilation of the critical frequency *fo*F2 of the F2 layer (*fo*F2 is directly related to *Nm*F2 by means of the relation *fo*F2 = (*Nm*F2/(1.24·10^4^))^1/2^, where *fo*F2 and *Nm*F2 are respectively expressed in [MHz] and [el/cm^3^]) and of the propagation factor *M*(3000)F2 routinely recorded by a network of European ionosondes. These assimilated characteristics are employed by IRI UP to calculate, at each ionosonde location, effective values *IG*_12eff_ and *R*_12eff_ of the ionospheric index *IG*_12_^15^ and of the solar index *R*_12_. Maps of such effective indices are then generated through the Universal Kriging method^16^, and are used as input for the IRI model to obtain updated values of *fo*F2 and *hm*F2 over the European region^13,14^. The application of the IRI UP method turned out to be very efficient in obtaining more reliable F2-layer characteristics for both quiet and magnetically disturbed conditions^13,14^.

The IRI UP method, assimilating data recorded with a fifteen minutes repetition rate by fourteen European ionosondes (see Table 1 of Pignalberi, *et al*.^13^), outputs updated *fo*F2 and *hm*F2 maps over the European region (from 15°W to 45°E in longitude and from 30°N to 60°N in latitude, with a 1° × 1° spatial resolution) with a periodicity of fifteen minutes. IRI UP *fo*F2 and *hm*F2 maps have been calculated for each Swarm satellites’ passage over the European region from 5 December 2013 to 30 September 2018. Ionosonde data used in this study were downloaded from the Digital Ionogram DataBASE^17^ by means of the SAO Explorer software developed by the University of Massachusetts, Lowell, and were autoscaled by two different systems: ARTIST (Automatic Real-Time Ionogram Scaler with True height analysis)^18,19^ and Autoscala^20,21^.

### Swarm’s satellite constellation data

Swarm is a satellite constellation constituted by three Low Earth Orbit satellites (named A, B, and C) launched at the end of 2013 by the European Space Agency (ESA)^22,23^. Main scopes of the Swarm mission is studying the geomagnetic field, the electric currents in the magnetosphere and ionosphere, and the impact of the solar wind on the dynamics of the upper atmosphere.

Swarm satellites are orbiting the Earth in a circular near-polar orbit; specifically, Swarm A and C fly side-by-side at the same altitude of about 460 km (with an inclination of 87.4°, an east-west separation of 1°–1.5° in longitude, and a maximal differential delay in orbit of approximately 10 seconds), while Swarm B flies at an altitude of about 520 km (with an inclination of 88°) in an orbital plane which has gradually got farther away from those of the other two satellites during the mission’s lifetime (9 hours in local time after 4 years).

All Swarm satellites are equipped with identical instruments consisting of GPS receivers and high-resolution sensors for measurements of both magnetic and electric fields, and plasma density.

In this work we used: (a) Level 1b electron density measurements at 2 Hz rate made by Langmuir probes^24–26^; (b) Level 2 TEC measurements made by GPS receivers at 0.1 Hz at the beginning of the mission, and at 1 Hz from 16 July 2014 onwards^27,28^. Swarm’s data are freely downloadable at ftp://swarm-diss.eo.esa.int.

In a first stage, Swarm’s electron density measurements are used to model the NeQuick *H*_0_ parameter over the European region. In a second stage, Swarm’s vTEC measurements are used to validate the new topside formulation.

Swarm Level 1b Langmuir probe data (EFIx_LP_1B) contain time series of corrected and calibrated electron density as observed along the satellites’ orbit. The EFIx_LP_1B dataset regularly undergoes upgrades leading to significant improvements in the data quality (https://earth.esa.int/web/guest/swarm/data-access/quality-of-swarm-l1b-l2cat2-products); such data quality improvements define different Product Baselines identified by two digits in the EFIx_LP_1B data filename. Electron density data used in this work concern the Latest Baseline, i.e. the 05, for which no calibration, like the one proposed by Lomidze *et al*.^25^, has been applied.

Swarm’s electron density data are however provided with two flags, one indicating the source of measurements (*Flags_LP*), the other characterizing the plasma density measurements (*Flags_Ne*), as reported in the Swarm L1b Product Definition^26^. For the development of the proposed topside model only the most reliable measurements, those with *Flags_LP* = 1 and *Flag_Ne* ≤ 29, were selected, from 5 December 2013 to 30 September 2018.

Since the IRI UP method provides F2-layer peak data over the European region, Swarm measurements were gridded over the same region, for every passage of Swarm satellites, and for the whole period under study, according to Pignalberi, *et al*.^5^. In particular, for a definite passage of a Swarm satellite over Europe, all electron density measurements falling in a 1° × 1° grid point were collected and undergone to the Median Absolute Deviation (MAD) filtering^25,29^, to remove any possible outlier. After that, the mean value of electron density measurements inside each grid point was calculated. These gridded mean electron density values were used as topside anchor points for the calculation of NeQuick *H*_0_ parameter values.

Differently, Swarm TEC data^27^ are not provided with any flag. Anyhow, the relative sTEC RMSE (Root Mean Square Error of relative slant TEC) is provided along with absolute vTEC, absolute sTEC, and relative sTEC measurements. Knowing the relative sTEC value along with the relative sTEC RMSE value, a relative percentage has been calculated as

1 $${\rm{sTEC}}\,\text{RMSE}\,[ \% ]=|\frac{{\rm{relative}}\,{\rm{sTEC}}\,{\rm{RMSE}}}{{\rm{relative}}\,{\rm{sTEC}}}|\cdot 100;$$

vTEC data for which sTEC RMSE > 5% have been discarded considering them as affected by excessive fluctuations. Moreover, as recommended in the Swarm L2 TEC product description^27^, only vTEC data with corresponding elevation angles ≥50° have been considered in this study. Hence, for a definite moment of time, vTEC values calculated for each GPS satellite in view have been MAD filtered and averaged in a 1° × 1° grid point to obtain a single vTEC value. vTEC data collected from 25 November 2013 to 30 September 2018 have been used to statistically test the new topside formulation proposed in this work. Specifically, Northern mid-latitude vTEC data between 30°N and 60°N in Quasi-Dipole magnetic coordinates^30^, and Southern mid-latitude vTEC data between 60°S and 30°S in Quasi-Dipole magnetic coordinates, have been considered.

### Improving the NeQuick topside formulation

The NeQuick topside analytical formulation^10,12^, which is one of the three topside options of the IRI model^7,8,11^, consists in a semi-Epstein layer describing the electron density *N*_e_ decrease in the topside region, as a function of the height *h*, starting from the *Nm*F2 value at the *hm*F2 height:

2.1 $${N}_{{\rm{e}}}(h)=4Nm{\rm{F2}}\frac{\exp (z)}{{(1+\exp (z))}^{2}};$$

the electron density decrease is driven by a reduced height *z*

2.2 $$z=\frac{h-hm{\rm{F2}}}{H},$$

dependent on a modeled scale height

2.3 $$H={H}_{0}[1+\frac{100\cdot 0.125(h-hm{\rm{F2}})}{100\cdot {H}_{0}+0.125(h-hm{\rm{F2}})}].$$

NeQuick models the scale height as a function of the empirically deduced parameter *H*_0_, which is dependent on *Nm*F2, *fo*F2, *M*(3000)F2, *hm*F2, and *R*_12_^11,12,31^. The parameter *H*_0_ reflects the scale height behavior at the F2-layer peak height *hm*F2 (*H* = *H*_0_ when *h* = *hm*F2, see Eq. (2.3)); then, the theoretically deduced (and experimentally proved) increase with height of the scale height is analytically modeled through Eq. (2.3).

The main idea behind this work is to improve the reliability of the scale height by calculating corrected values of the *H*_0_ parameter. To obtain a corrected value for *H*_0_, for a definite time and location, the NeQuick topside formula (2.1) is forced to join the F2-layer peak point (*hm*F2, *Nm*F2), provided by the IRI UP method, and the topside anchor point (*h*_sat_, *N*_e,sat_), provided by Swarm satellites. This operation is repeated for every couple of co-located and simultaneously measured/modeled anchor points constituting the dataset previously described, which allows the calculation of a huge amount of *H*_0_ values over the European region.

Calculated *H*_0_ values are modeled as a function of *fo*F2 and *hm*F2 and to accomplish this task *H*_0_ values calculated by using Swarm A and C datasets are joined, taking into account that these satellites fly at the same height very close to each other.

Specifically, *H*_0_ values are modeled as a function of *fo*F2 and *hm*F2 by applying the two-dimensional binning procedure described in Pignalberi, *et al*.^5^, briefly:

1. Each calculated value of *H*_0_ is associated to a definite pair (*fo*F2, *hm*F2);
2. *H*_0_ values are two-dimensional binned as a function of *fo*F2 and *hm*F2, with a bin width of 0.25 MHz and 5 km, respectively, for *fo*F2 ∈ [0, 16] MHz and *hm*F2 ∈ [150, 450] km;
3. The median of *H*_0_ values falling inside each bin is calculated, provided that the number of values is greater than or equal to 10, otherwise the bin is considered statistically not significant.

**Figure 1:**
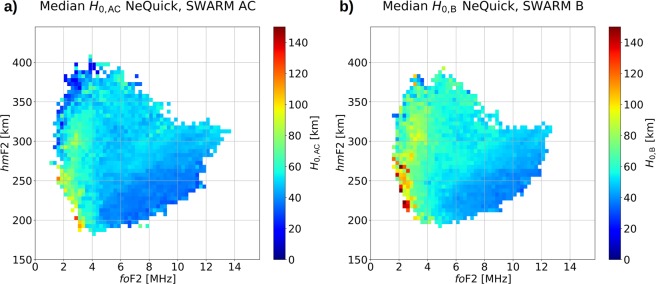
(**a**) Median values of *H*_0,AC_ calculated by using the NeQuick topside formulation, the IRI UP modeled values and Swarm A and C electron density measurements. (**b**) Median values of *H*_0,B_ calculated by using the NeQuick topside formulation, the IRI UP modeled values and the Swarm B electron density measurements. The median was calculated only for bins including a number of values greater than or equal to 10.

Figure 1 shows the two-dimensional binned grid of *H*_0_ calculated by using Swarm A and C datasets (whose values will be hereafter identified as *H*_0,AC_), and the two-dimensional binned grid calculated by using the Swarm B dataset (whose values will be hereafter identified as *H*_0,B_).

Once a pair (*fo*F2, *hm*F2) is defined (measured or modeled), the selection of the corresponding *H*_0_ value in the two-dimensional grids of *H*_0_ values of Fig. 1 allows to model the topside vertical electron density profile through Eq. (2.1–2.3). By means of the two-dimensional grids of Fig. 1, two different topside profiles are obtained by using *H*_0,AC_ and *H*_0,B_ in Eq. (2.3).

Pignalberi, *et al*.^5^ showed how scale heights modeled by using Swarm A and C datasets are more reliable than those modeled by using the Swarm B one. As expected, also in this case we have found different performances when using the two *H*_0_ grids of Fig. 1. In particular, we verified that the use of the *H*_0,AC_ grid gives better results from *hm*F2 to approximately the Swarm A and C altitude, while the use of the *H*_0,B_ grid gives better results from the Swarm B altitude to higher ones.

According to these results, for a definite pair (*fo*F2, *hm*F2), we implemented a corrected version of the *H*_0_ parameter, named *H*_0,corr_, as follows:

3.1 $$\begin{array}{lll}{H}_{0,{\rm{corr}}}={H}_{0,{\rm{AC}}} & {\rm{for}} & h=hm\text{F2},\end{array}$$

32 $$\{\begin{array}{ccc}{H}_{0,{\rm{c}}{\rm{o}}{\rm{r}}{\rm{r}}}={H}_{0,\mathrm{AC}}+({H}_{0,{\rm{B}}}-{H}_{0,\mathrm{AC}})\cdot \alpha  & {\rm{f}}{\rm{o}}{\rm{r}} & h\in (hm{\rm{F}}2,hm{\rm{F}}2+600)\\ \alpha =\frac{h-hm{\rm{F}}2}{600} &  & \end{array},$$

33 $$\begin{array}{ccc}{H}_{0,{\rm{c}}{\rm{o}}{\rm{r}}{\rm{r}}}={H}_{0,{\rm{B}}} & {\rm{f}}{\rm{o}}{\rm{r}} & h\ge hm{\rm{F}}2+600\end{array}.$$

Equation (3.1–3.3) show that *H*_0,corr_, starting from *H*_0,AC_ at *hm*F2, increases linearly towards *H*_0,B_ between *hm*F2 and 600 km above *hm*F2, and then assumes the constant value *H*_0,B_ for higher altitudes. For the very few bins for which *H*_0,B_ is lower than or equal to *H*_0,AC_, we assume *H*_0,corr_ always constant and equal to *H*_0,AC_ for *h* > *hm*F2. If only one value of either *H*_0,AC_ or *H*_0,B_ is available for a definite (*fo*F2, *hm*F2) pair, this is assumed to be equal to *H*_0,corr_ for *h* > *hm*F2. Instead, the original NeQuick *H*_0_ parameter has to be used for those cases for which neither *H*_0,AC_ nor *H*_0,B_ is available.

**Figure 2:**
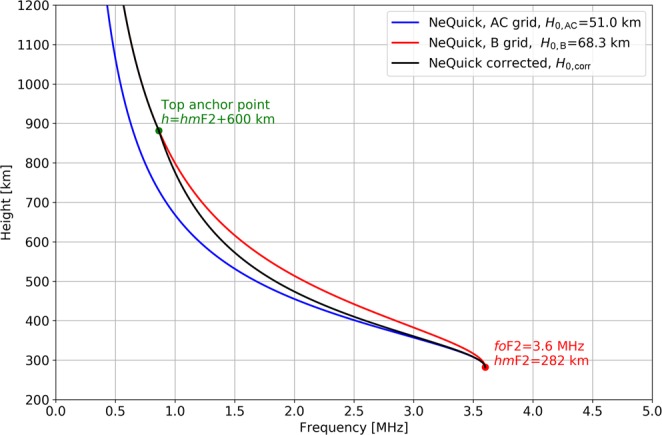
Topside vertical electron density profiles as modeled by applying the NeQuick topside formulation using (blue) *H*_0,AC_, (red) *H*_0,B_, and (black) *H*_0,corr_ instead of *H*_0_. The red dot identifies the F2-layer peak (*fo*F2 = 3.6 MHz, *hm*F2 = 282 km), where *H*_0,corr_ = *H*_0,AC_, while the green dot identifies the point at which *h* = *hm*F2 + 600 km and *H*_0,corr_ = *H*_0,B_.

Figure 2 shows an example of topside vertical electron density profiles as modeled by using *H*_0,AC,_ *H*_0,B_, and *H*_0,corr_, for the pair (*fo*F2 = 3.6 MHz, *hm*F2 = 282 km). Looking at Fig. 2, we can see how using *H*_0,corr_ the NeQuick profile is very similar to the one modeled by using *H*_0,AC_ immediately above the F2-layer peak, and then smoothly reaches the one modeled by using *H*_0,B_ at the height *h* = 882 km (because this is the altitude at which *h* = *hm*F2 + 600 km). Choosing *h* = *hm*F2 + 600 km as the height at which *H*_0,corr_ = *H*_0,B_ has been empirically validated in a preliminary testing phase.

### COSMIC/FORMOSAT-3 radio occultation data

Constellation Observing System for Meteorology, Ionosphere, and Climate (COSMIC)^32^, also known as FORMOSAT-3 in Taiwan, is a joint project between the National Space Organization in Taiwan and the University Corporation for Atmospheric Research in the United States. In the framework of this project, six microsatellites were launched in April 2006 into a circular orbit (with 72° of inclination) at about 800 km of height, with a separation angle of 30° in longitude between them.

Each satellite carries a GPS Radio Occultation (RO) receiver able to measure the phase delay of radio waves from GPS satellites as they are occulted by the Earth’s atmosphere, allowing an accurate determination of the ionospheric vertical electron density profile. COSMIC RO profiles are obtained by means of the Abel inversion technique^33,34^, assuming the spherical symmetry hypothesis. Such simplifying hypothesis can produce errors when strong horizontal electron density gradients are present, like in the Equatorial Ionization Anomaly region, during dawn and dusk hours, and during intense geomagnetically disturbed events^35,36^.

COSMIC RO “*ionprf*   ” files, containing the ionospheric electron density profile, were downloaded by means of the COSMIC Data Analysis and Archive Center (http://cdaac-www.cosmic.ucar.edu/cdaac/products.html). COSMIC data from 22 April 2006 to 30 September 2018 whose F2-layer tangent point falls inside the Northern mid-latitude region, from 30°N to 60°N in Quasi-Dipole magnetic coordinates^30^, or inside the Southern mid-latitude region, from 30°S to 60°S in Quasi-Dipole magnetic coordinates, have been used to validate the proposed topside model. COSMIC data showed a pretty good reliability in the description of both the topside ionosphere and the underlying F-region^36–41^. Anyhow, COSMIC RO profiles showing *fo*F2 and *hm*F2 values outside the range [1, 16] MHz and [150, 450] km were discarded, as well as profiles with excessive and unrealistic fluctuations in the topside electron density. RO derived profiles are inherently slanted, while the proposed topside model produces vertical electron density profiles. This means that RO electron density values for different altitudes above *hm*F2 refer to different geographical locations that are not along a vertical axis. In some cases, this difference can be very important and can lead to significant errors. To limit this issue, only RO profiles observing the following specifications have been considered:

4 $$\begin{array}{c}|La{t}_{hm{\rm{F2}}}-La{t}_{600{\rm{km}}}|\le 5^\circ \\ |Lo{n}_{hm{\rm{F2}}}-Lo{n}_{600{\rm{km}}}|\le 10^\circ ,\end{array}$$

where *Lat*_*hm*F2_ and *Lat*_600km_ are the geographic latitudes, and *Lon*_*hm*F2_ and *Lon*_600km_ are the geographic longitudes, of electron density values recorded at *hm*F2 and 600 km of height, respectively. This range of altitude, between *hm*F2 and 600 km, is the one used to calculate RO vTEC values. We conclude this section by saying that recently Cherniak and Zakharenkova^42^, using COSMIC RO data, have shown that the NeQuick specification significantly misrepresents the electron content of the topside ionosphere region, independently of solar activity.

## Results and Validation

The procedure described in the “Improving the NeQuick topside formulation” subsection allows for modeling the topside vertical electron density profile once *fo*F2 and *hm*F2 ionospheric characteristics are available, either measured or modeled. Specifically:

1. *H*_0,AC_ and *H*_0,B_ values are picked from grids of Fig. 1a,b according to a measured or modeled (*fo*F2, *hm*F2) pair;
2. The *H*_0,corr_ parameter is calculated through Eq. (3.1–3.3);
3. The NeQuick topside scale height *H* is calculated through Eq. (2.3);
4. A topside vertical electron density profile is calculated through Eq. (2.1) by using the scale height obtained at point (3).

For the sake of simplicity, the new topside formulation is called *NeQuick corrected* (hereafter *NeQuick-corr*).

The NeQuick-corr topside formulation was evaluated in terms of residuals and RMSE values, both expressed in TECU (1 TECU = 10^16^el/m^2^), by comparing corresponding vTEC values with those provided by COSMIC RO profiles and measured by Swarm satellites, according to the following relationships:

5 $${\rm{vTEC}}\,{\rm{residuals}}\,[\text{TECU}]\,={{\rm{vTEC}}}_{\mathrm{mod}{\rm{eled}}}-{{\rm{vTEC}}}_{{\rm{measured}}},$$

6 $${\rm{RMSE}}\,[\text{TECU}]\,=\sqrt{\frac{{{\sum }_{i=1}^{N}({{\rm{vTEC}}}_{\mathrm{mod}\text{eled},i}-{{\rm{vTEC}}}_{\text{measured},i})}^{2}}{N},}$$

where *N* is the number of values. vTEC_modeled_ values are those modeled by both the NeQuick-corr procedure and the original NeQuick topside formulation^12^ (hereafter IRI-NeQuick), the latter being one of the three topside options proposed by IRI^6^. vTEC_measured_ values are either those obtained by integrating the RO profiles or those measured by Swarm.

vTEC values calculated from COSMIC RO profiles allow for a validation of the lower topside region, while Swarm measured vTEC values allow for a validation of the upper topside region. Although the proposed procedure was developed by using data collected over the European region (a constraint related to the fact that grids of Fig. 1 have been calculated with the help of the IRI UP method), a validation for the two mid-latitudes bands, between 30°N and 60°N and between 30°S and 60°S, in Quasi-Dipole magnetic coordinates, has been performed to verify its applicability for regions different from Europe.

### Validation with COSMIC topside vTEC

**Figure 3:**
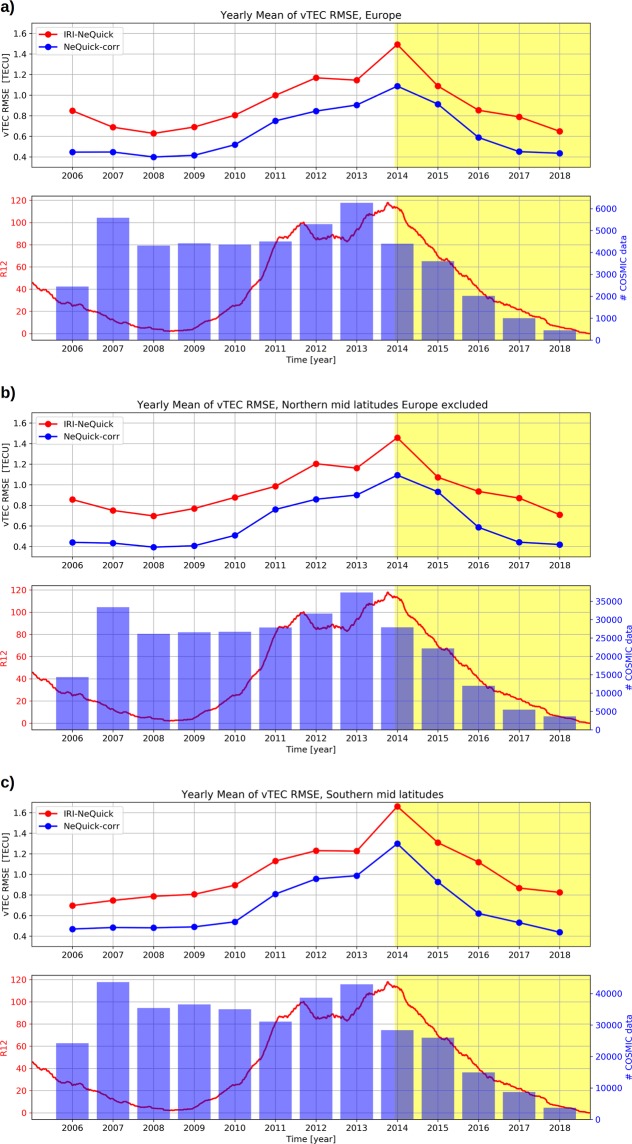
Yearly mean of RMSE between (red) the IRI-NeQuick model and COSMIC derived vTEC values and between (blue) the NeQuick-corr model and COSMIC derived vTEC values, for: (**a**) the European region; (**b**) Northern mid latitudes Europe excluded; (**c**) Southern mid latitudes. In each panel, the *R*_12_ solar activity index as well as the number of COSMIC RO data considered for each year are reported. Years highlighted in yellow are those covered by the Swarm mission.

COSMIC RO profiles from 22 April 2006 to 30 September 2018 for the aforementioned Northern and Southern mid-latitude bands are used for this validation. The number of data used, for each year and for both mid-latitude sectors, is shown in Fig. 3.

For each RO profile, vTEC is calculated by integrating topside electron density values from *hm*F2 to 600 km, as follows:

7 $${{\rm{v}}{\rm{T}}{\rm{E}}{\rm{C}}}_{{\rm{m}}{\rm{e}}{\rm{a}}{\rm{s}}{\rm{u}}{\rm{r}}{\rm{e}}{\rm{d}}}={\int }_{hm{\rm{F}}2}^{600}{N}_{\text{e},{\rm{C}}{\rm{O}}{\rm{S}}{\rm{M}}{\rm{I}}{\rm{C}}}{\rm{d}}h.$$

Moreover, each topside RO profile provides measured values of *fo*F2 and *hm*F2 that are used to model the topside profile by following the procedure described in the “Improving the NeQuick topside formulation” subsection and at the beginning of the “Results and validation” section. vTEC modeled values are then calculated as follows:

8 $${{\rm{v}}{\rm{T}}{\rm{E}}{\rm{C}}}_{{\rm{m}}{\rm{o}}{\rm{d}}{\rm{e}}{\rm{l}}{\rm{e}}{\rm{d}}}={\int }_{hm{\rm{F}}2}^{600}{N}_{\text{e},{\rm{m}}{\rm{o}}{\rm{d}}{\rm{e}}{\rm{l}}{\rm{e}}{\rm{d}}}{\rm{d}}h.$$

vTEC_modeled_ values are those modeled by both the NeQuick-corr procedure and IRI-NeQuick. The IRI-NeQuick topside profiles were calculated by ingesting *fo*F2 and *hm*F2 values measured by COSMIC. In this way, vTEC_modeled_ values and vTEC_measured_ values are based on the same F2-layer peak anchor point.

In Fig. 3, the yearly mean of calculated RMSE between vTEC values modeled by the NeQuick-corr procedure and measured by COSMIC, and between vTEC values modeled by IRI-NeQuick and measured by COSMIC, have been calculated for three regions: (a) Europe, from 30°N to 60°N and from 15°W to 45°E in geographical coordinates; (b) Northern mid latitudes not including Europe, from 30°N to 60°N in Quasi-Dipole magnetic coordinates; (c) Southern mid latitudes, from 30°S to 60°S in Quasi-Dipole magnetic coordinates. Years from 2006 to 2018 comprise the last part of solar cycle 23, including the prolonged and peculiar minimum characterizing it^43,44^, and the whole solar cycle 24, as depicted in Fig. 3 by the trend of solar index *R*_12_.

Motivations for the validation analysis shown in Fig. 3 are threefold:

1. Verifying if the NeQuick-corr formulation can improve the original NeQuick formulation;
2. Verifying if the NeQuick-corr formulation can be profitably applied also outside the European region, and in particular at both Northern and Southern mid latitudes;
3. Verifying if the NeQuick-corr formulation is reliable also for time windows outside that, from December 2013 to September 2018, used for its implementation.

Point (1) is evaluated by comparing RMSE yearly mean values for NeQuick-corr with those for IRI-NeQuick, and Fig. 3 shows that NeQuick-corr significantly improves the IRI-NeQuick output.

Point (2) is evaluated by looking at NeQuick-corr results for different geographical regions shown in Fig. 3: Europe in panel (a), Northern mid latitudes but excluding the European region in panel (b), and Southern mid latitudes in panel (c). Figure 3 shows that NeQuick-corr performances are very similar, independently of the region, despite only Swarm and IRI UP data for the European region were employed for the development of the NeQuick-corr formulation. This suggests that the NeQuick-corr model can be reliably used for mid latitudes without any restriction in longitude.

Point (3) is evaluated by looking at NeQuick-corr performances for different years. As described in the “Data and method” section, for the development of the NeQuick-corr formulation, only data from December 2013 to September 2018 were used. Nevertheless, Fig. 3 shows that NeQuick-corr can improve IRI-NeQuick performances also for years outside this time window, specifically from 2006 to the end of 2013. It is important to highlight that the dataset used to derive the NeQuick-corr formulation spans from high solar activity (year 2014) to low solar activity (year 2018), so the proposed formulation is able to describe the topside ionosphere for both solar activity conditions.

**Figure 4:**
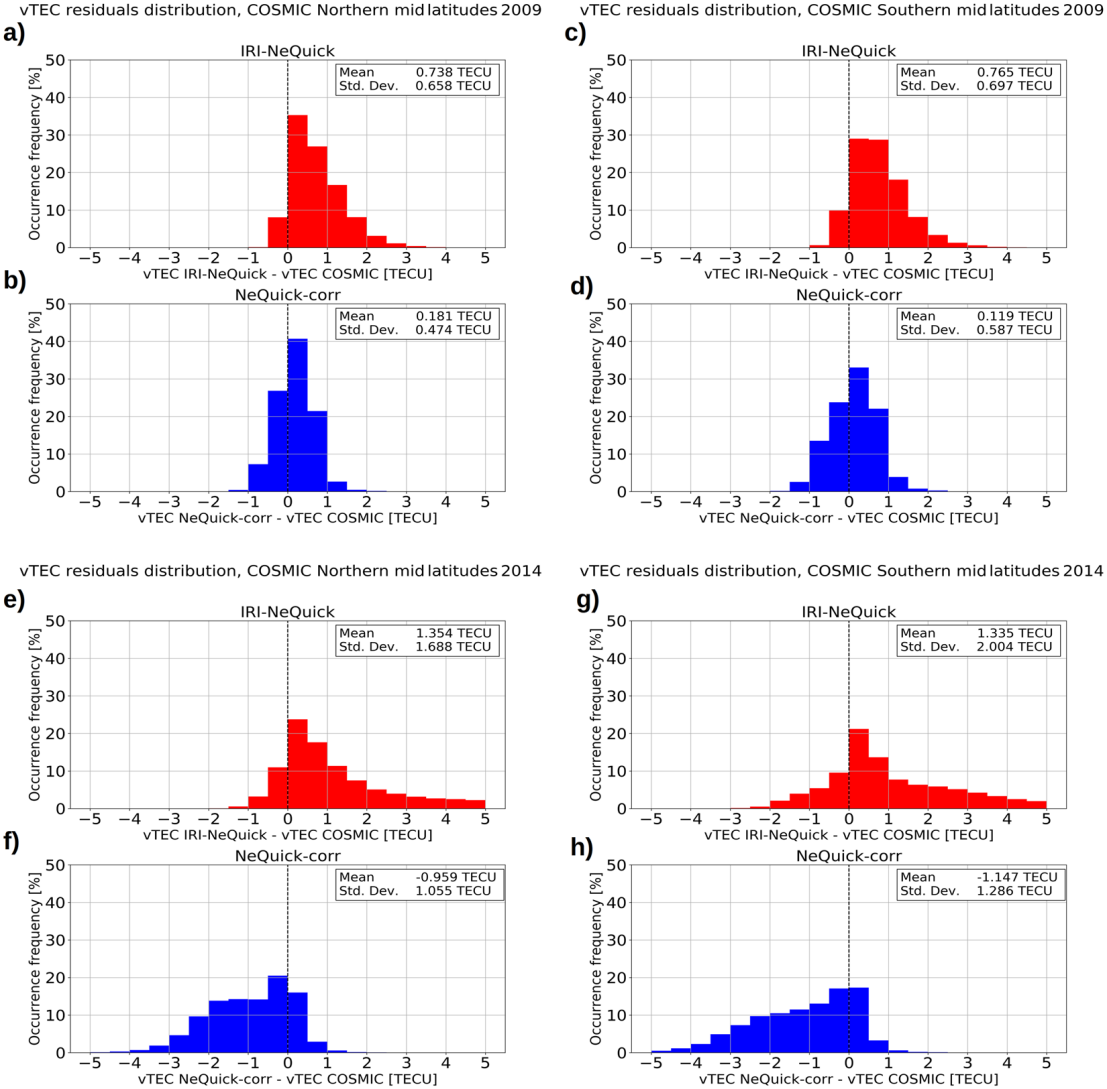
Statistical distributions of residuals between vTEC values derived from COSMIC RO profiles and vTEC values as modeled by (red) IRI-NeQuick and by (blue) the proposed NeQuick-corr topside model. Panels (a,b) refer to Northern mid latitudes for year 2009 (low solar activity). Panels (c,d) refer to Southern mid latitudes for year 2009. Panels (e,f) refer to Northern mid latitudes for year 2014 (high solar activity). Panels (g,h) refer to Southern mid latitudes for year 2014.

RMSE values represented in Fig. 3 give an overall information about the error done in vTEC modeling; instead, vTEC residuals distributions for different years can highlight whether modeled values are overestimating or underestimating measured values. With regard to this, Fig. 4 shows the residuals distributions between modeled and measured values by COSMIC for both year 2009 (low solar activity) and year 2014 (high solar activity). Residual distributions clearly show that IRI-NeQuick performs an overestimation for both Northern and Southern mid latitudes and for different solar activities, according to Cherniak and Zakharenkova^42^. Differently, NeQuick-corr slightly overestimates measured values for low solar activity (year 2009) and tends to underestimate measured values for high solar activity (year 2014), for both the Northern and the Southern mid-latitude band.

The analysis done for vTEC in Fig. 3 was also performed by comparing the whole electron density topside profiles, modeled and measured (see Supplementary Fig. S1). Corresponding results are very similar.

### Validation with Swarm vTEC values

**Figure 5:**
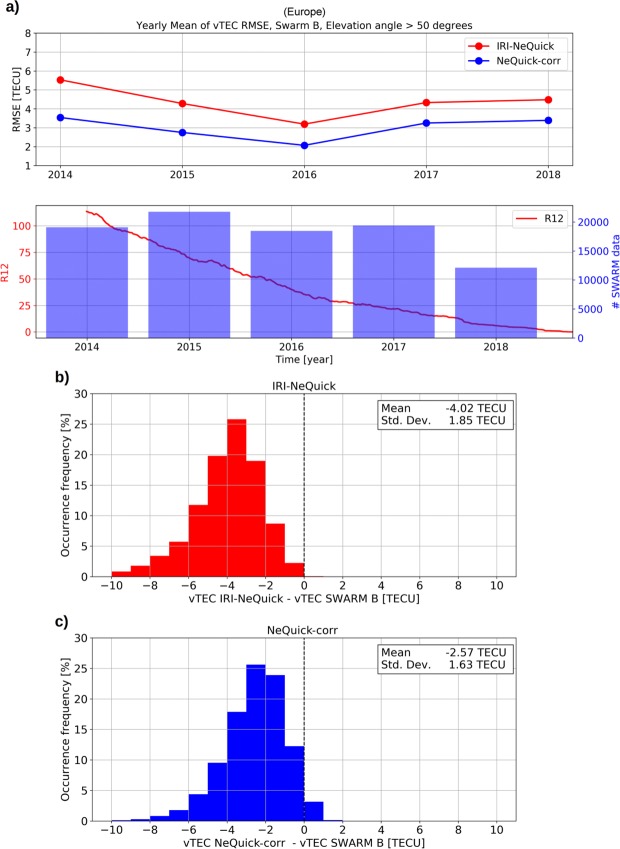
(**a**) Yearly mean of RMSE between (red) IRI-NeQuick vTEC derived values and Swarm B vTEC values and between (blue) NeQuick-corr vTEC derived values and Swarm B vTEC values, for the European region; both IRI-NeQuick and NeQuick-corr were fed with *fo*F2 and *hm*F2 provided by IRI UP. The *R*_12_ solar activity index trend as well as the number of Swarm B data for each considered year are also reported. (**b**,**c**) Statistical distribution of corresponding residuals for (red) IRI-NeQuick and (blue) NeQuick-corr. Concerning vTEC values by Swarm, only GPS satellites with an elevation angle greater than 50 degrees were considered.

vTEC values measured by Swarm satellites from 5 December 2013 to 30 September 2018 are also considered to validate the NeQuick-corr topside formulation. Unlike values from COSMIC used in the “Validation with COSMIC topside vTEC” subsection, vTEC values measured by Swarm satellites refer to the upper topside region, from the Swarm orbit altitude (about 460 km for both Swarm A and C, and about 520 km for Swarm B) to the GPS satellites altitude. Unlike the COSMIC constellation, Swarm satellites cannot provide measurements of *fo*F2 and *hm*F2, which are needed to model the topside profile according to the procedure described in the “Improving the NeQuick topside formulation” subsection and at the beginning of the “Results and validation” section. For this reason, in this case, *fo*F2 and *hm*F2 values are provided by the IRI UP method over the European region and by the IRI model elsewhere. Statistical quantities (5) and (6) were calculated, where, in this case, vTEC_measured_ refers to values measured by Swarm satellites and vTEC_modeled_ values are calculated as:

9 $${{\rm{v}}{\rm{T}}{\rm{E}}{\rm{C}}}_{{\rm{m}}{\rm{o}}{\rm{d}}{\rm{e}}{\rm{l}}{\rm{e}}{\rm{d}}}={\int }_{{h}_{{\rm{S}}{\rm{w}}{\rm{a}}{\rm{r}}{\rm{m}}}}^{{h}_{{\rm{G}}{\rm{P}}{\rm{S}}}}{N}_{{\rm{e}},{\rm{m}}{\rm{o}}{\rm{d}}{\rm{e}}{\rm{l}}{\rm{e}}{\rm{d}}}{\rm{d}}h,$$

where *h*_Swarm_ and *h*_GPS_ are respectively the height of the Swarm orbit and the height of the GPS satellite orbit, which is here considered equal to 20200 km. Results obtained by using, for validation, vTEC values collected over the European region by Swarm B satellite are shown in Fig. 5. For both NeQuick-corr and IRI-NeQuick, vTEC values were calculated according to (9). Concerning IRI-NeQuick, in order to calculate corresponding vTEC values as the integral of the vertical electron density profile, we forced the upper boundary of the IRI model, usually set to 2000 km, to 20200 km; specifically, we considered the analytical formulation of NeQuick represented by Eq. (2.1–2.3), the one used by IRI as one of the three topside options, and we simply set its upper boundary to 20200 km.

According to Fig. 3, also Fig. 5 shows that NeQuick-corr significantly improves the IRI-NeQuick output, for all considered years, and independently of solar activity. At the same time, Fig. 5 shows that both topside models heavily underestimate measured values, according to Cherniak and Zakharenkova^42^.

**Figure 6:**
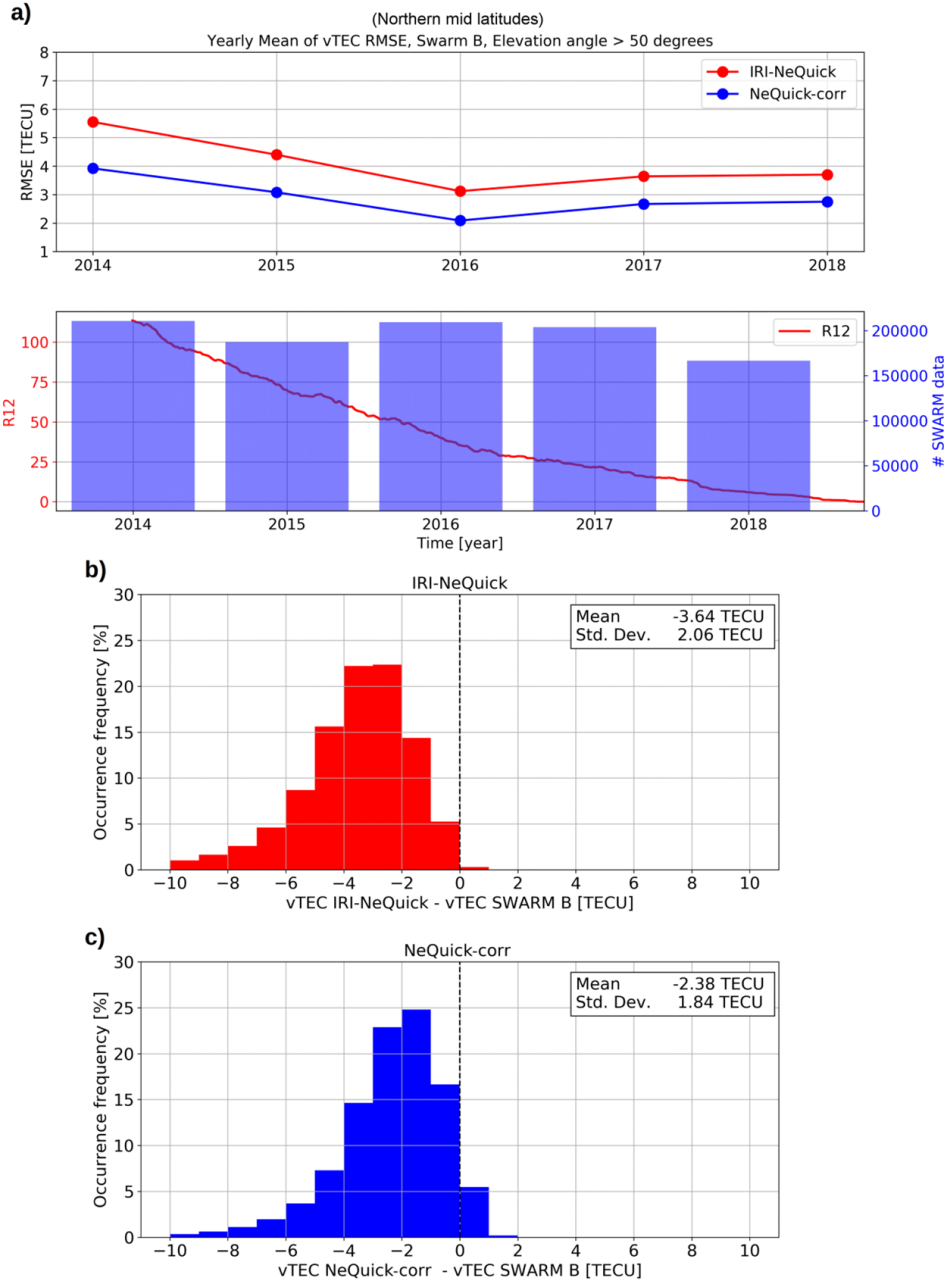
Same as Fig. 5 but for Northern mid latitudes (Europe included) and by using the IRI model for *fo*F2 and *hm*F2.

**Figure 7:**
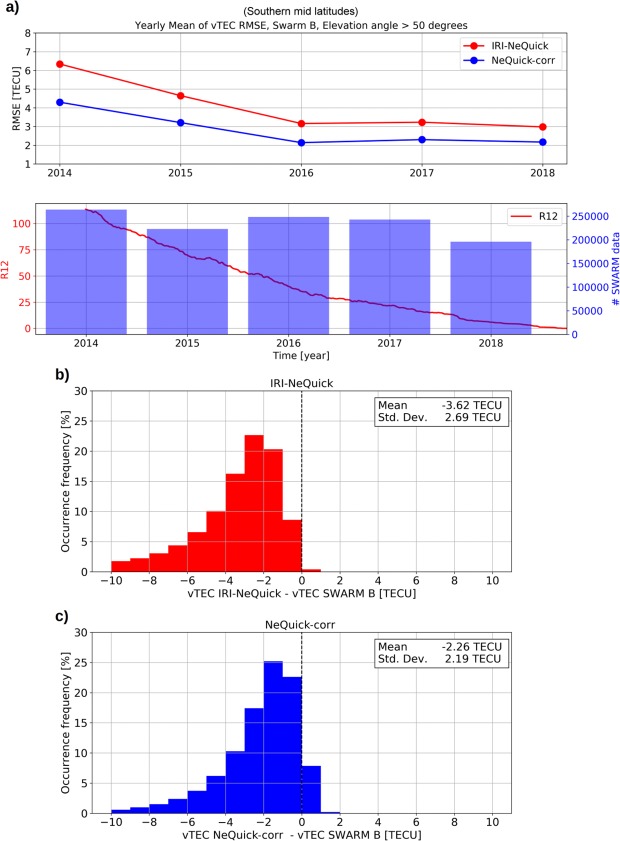
Same as Fig. 6 but for Southern mid latitudes.

To evaluate the NeQuick-corr formulation also for regions outside Europe, Swarm vTEC data have been considered for the aforementioned Northern and Southern mid-latitude bands. In this case, *fo*F2 and *hm*F2 values have been provided by the IRI model^6^ (considering the URSI option for *fo*F2 and the Shubin, *et al*.^45^ option for *hm*F2). Results are shown in Figs 6 and 7 for Northern mid latitudes (Europe included) and for Southern mid latitudes, respectively, by using Swarm B data, and confirm what already highlighted by Fig. 5. Results obtained by using Swarm A and C satellites are very similar to those characterizing Swarm B (see Supplementary Figs S2, S3 and S4 for Swarm A, and Supplementary Figs S5, S6 and S7 for Swarm C).

## Conclusions

In this work we have faced the challenging issue of modeling the topside part of the ionosphere. Specifically, we have considered the NeQuick formulation, which is the most used and reliable topside option among those proposed by the IRI model, and we tried to improve it by exploiting the valuable amount of *in-situ* electron density measurements made available by the ESA Swarm mission launched in November 2013. To accomplish this task, on the base of such data, we implemented a new formulation of the crucial parameter *H*_0_, used by NeQuick to model the scale height, and we validated it with both COSMIC RO data and TEC Swarm values. The results show that the new NeQuick formulation improves significantly the topside ionospheric representation, even though the important underestimation for the topside ionospheric and plasmaspheric regions made by the previous version of the model is strongly reduced but not eliminated; this is an issue to be explored in the near future. Moreover, although the new NeQuick formulation was implemented by considering data recorded over the European region, its performances are reliable also for two large mid-latitude bands in both hemispheres. It is the intention of the authors to test this new formulation also for high- and low-latitude regions, and in case to face and smooth possible shortcomings of the formulation, should they arise. The proposed two-dimensional binning of *H*_0_, as a function of only *fo*F2 and *hm*F2, lets the new proposed NeQuick formulation to be applied in the same way either to ionosonde’s derived measurements and to ionospheric models. Given the obtained results, the new NeQuick formulation might be proposed to be included in the next version of the IRI model. The two-dimensional grids of *H*_0,AC_ and *H*_0,B_, as a function of *fo*F2 and *hm*F2, are available as Supplementary Data S1 and S2.

## Supplementary information

Supplementary Figures

Supplementary Data S1

Supplementary Data S2
