# Publisher Correction: The ESA Swarm mission to help ionospheric modeling: a new NeQuick topside formulation for mid-latitude regions

**DOI:** 10.1038/s41598-020-61556-4

**Published:** 2020-03-10

**Authors:** M. Pezzopane, A. Pignalberi

**Affiliations:** Istituto Nazionale di Geofisica e Vulcanologia, Via di Vigna Murata 605, 00143 Rome, Italy

Correction to: *Scientific Reports* 10.1038/s41598-019-48440-6, published online 22 August 2019

Two supplementary files S1 and S2, containing the two-dimensional grids of *H*_0,AC_ and *H*_0,B_ as a function of *fo*F2 and *hm*F2 were omitted from the original version of this Article. This has been corrected in the HTML version of the Article; the PDF version was correct at time of publication.

