# Supplementary material for: The ESA Swarm mission to help ionospheric modeling: a new NeQuick topside formulation for mid-latitude regions
Source: Sci Rep. 2019 Aug 22;9:12253. doi: 10.1038/s41598-019-48440-6 (PMC6706453; doi:10.1038/s41598-019-48440-6)
Supplement: Supplementary file 1 — Supplementary Figures [file 41598_2019_48440_MOESM1_ESM.docx]

**The ESA Swarm mission to help ionospheric modeling: a new NeQuick topside formulation for mid-latitude regions**

**M. Pezzopane^1^ and A. Pignalberi^1^**

^1^Istituto Nazionale di Geofisica e Vulcanologia, Via di Vigna Murata 605, 00143, Rome, Italy.


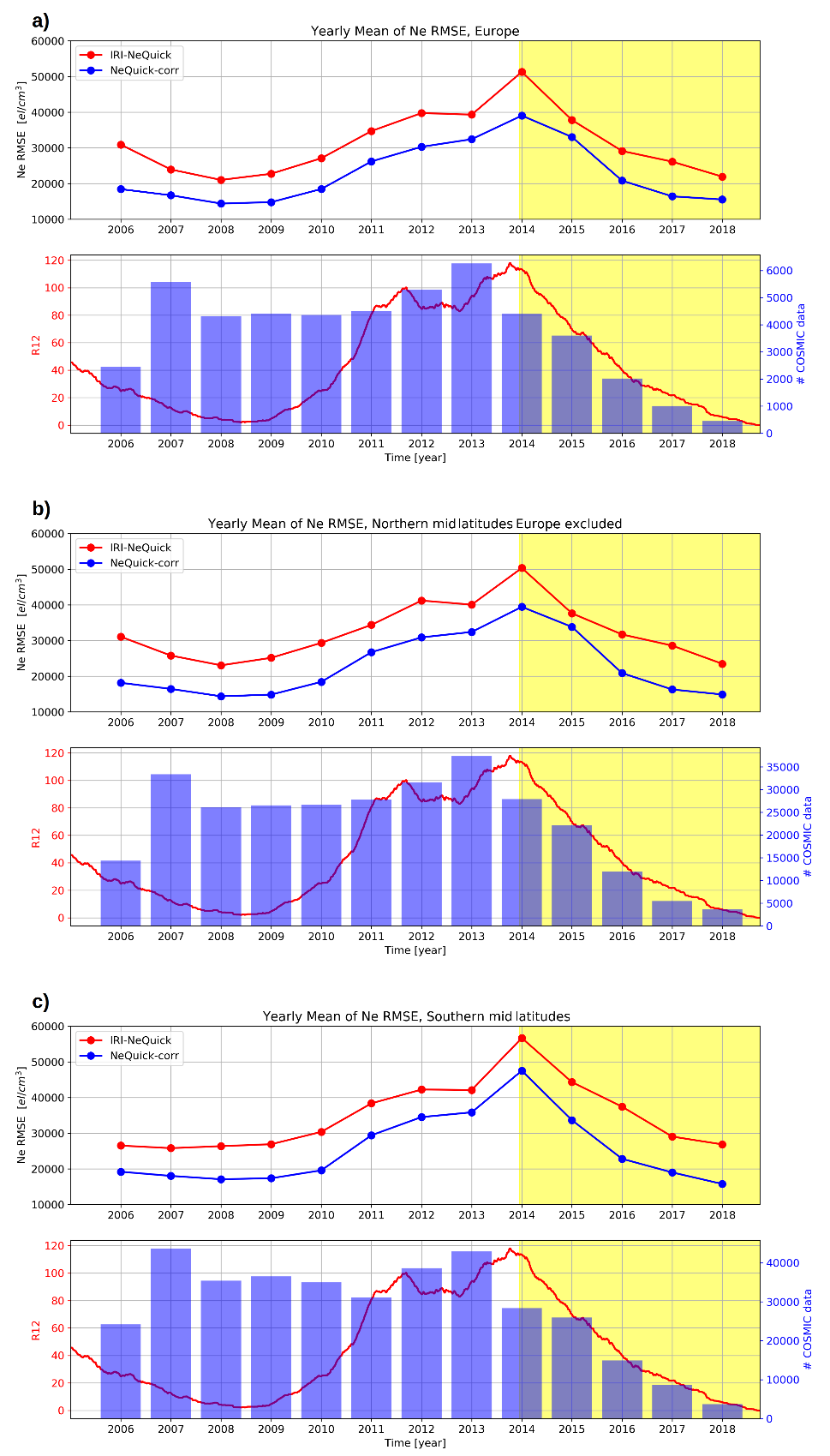


**Figure S1.** Same as Fig. 3 of the main paper but for *N*_e_. In this case, Eq. (6) is applied for each pair of whole topside profiles (modeled and measured), by considering *N*_e_ values every 1 km. Values of RSME obtained for each pair of profiles of a definite year were then averaged.


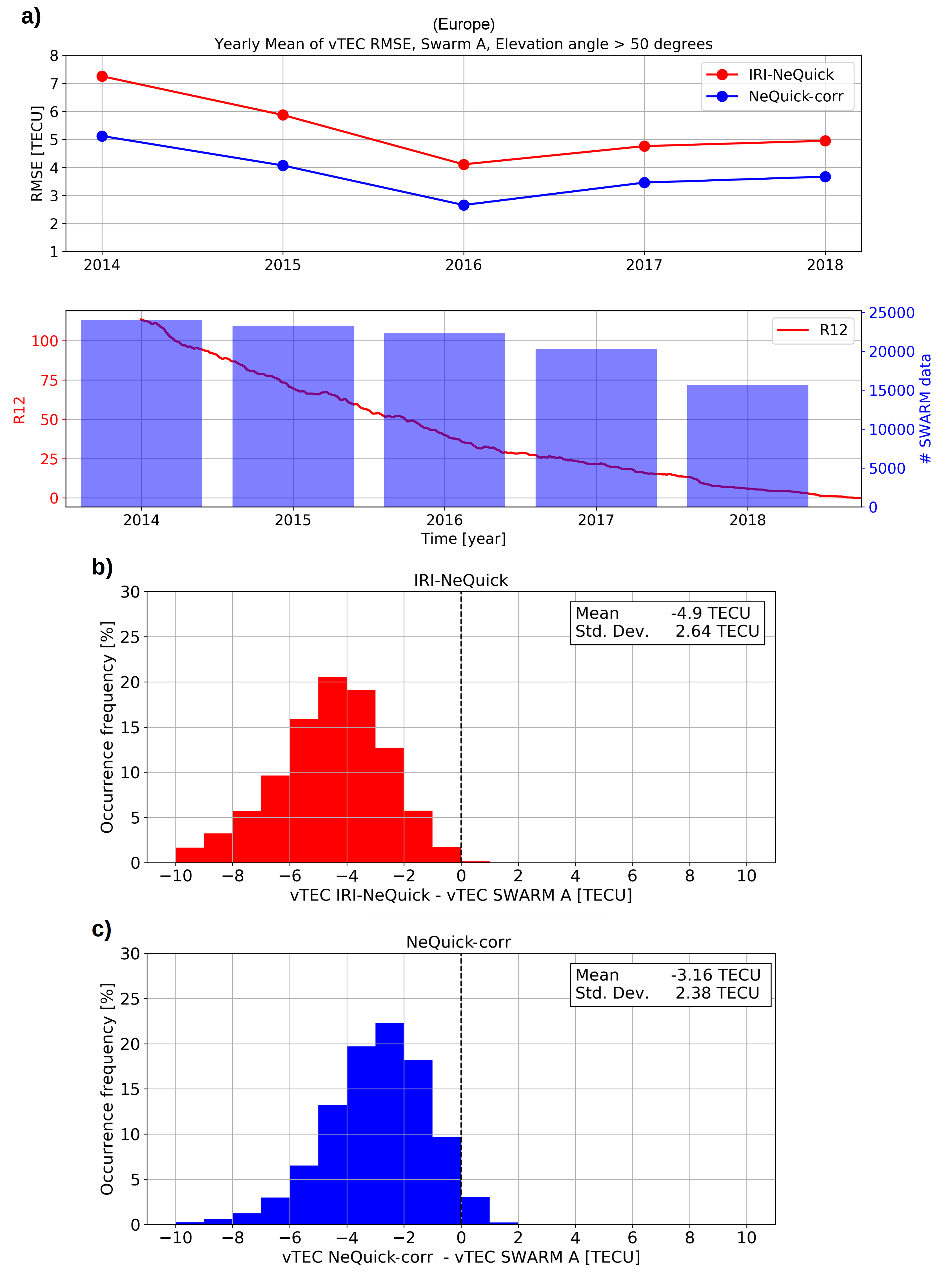


**Figure S2.** Same as Fig. 5 of the main paper but for Swarm A.


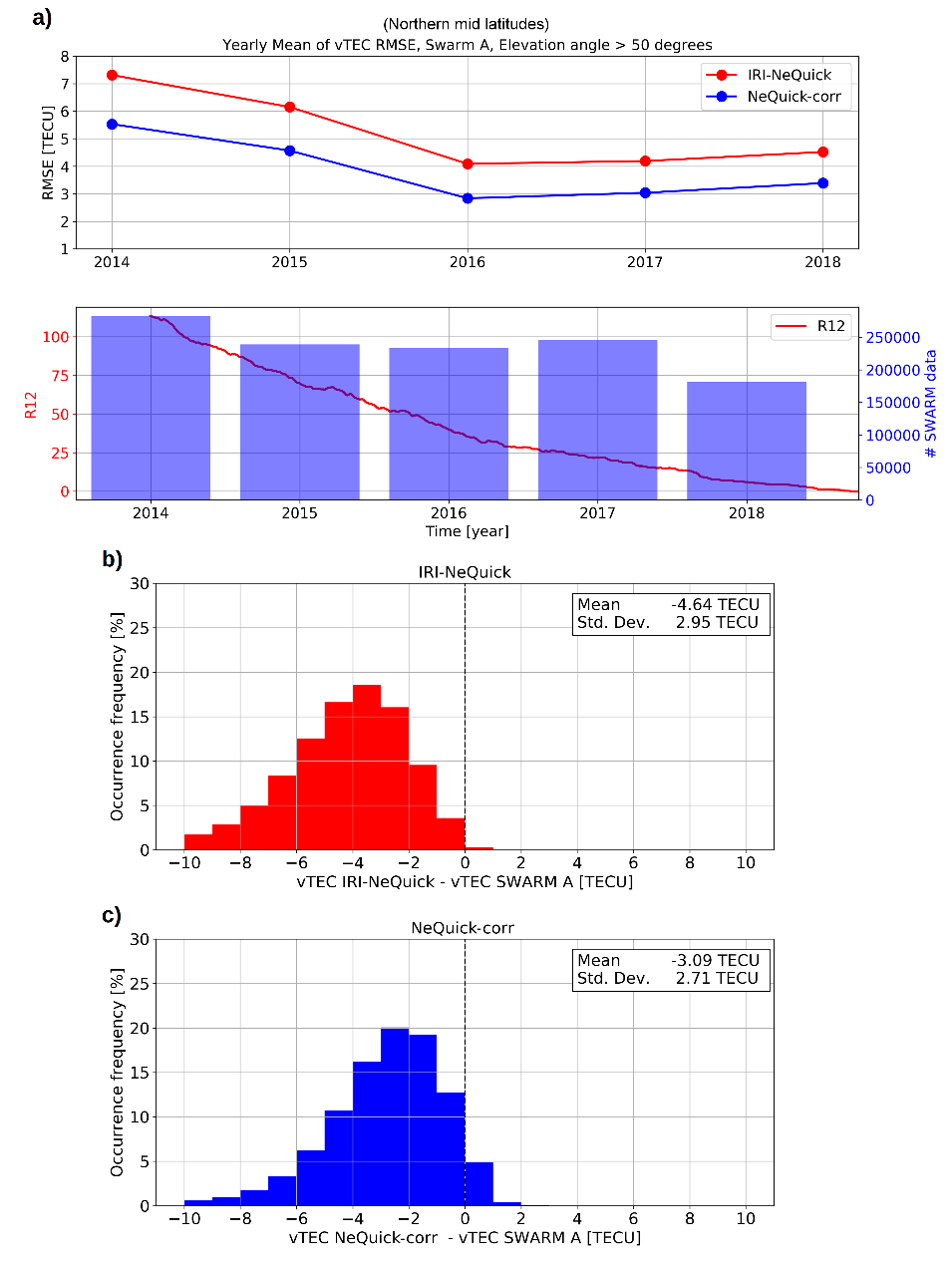


**Figure S3.** Same as Fig. 6 of the main paper but for Swarm A.


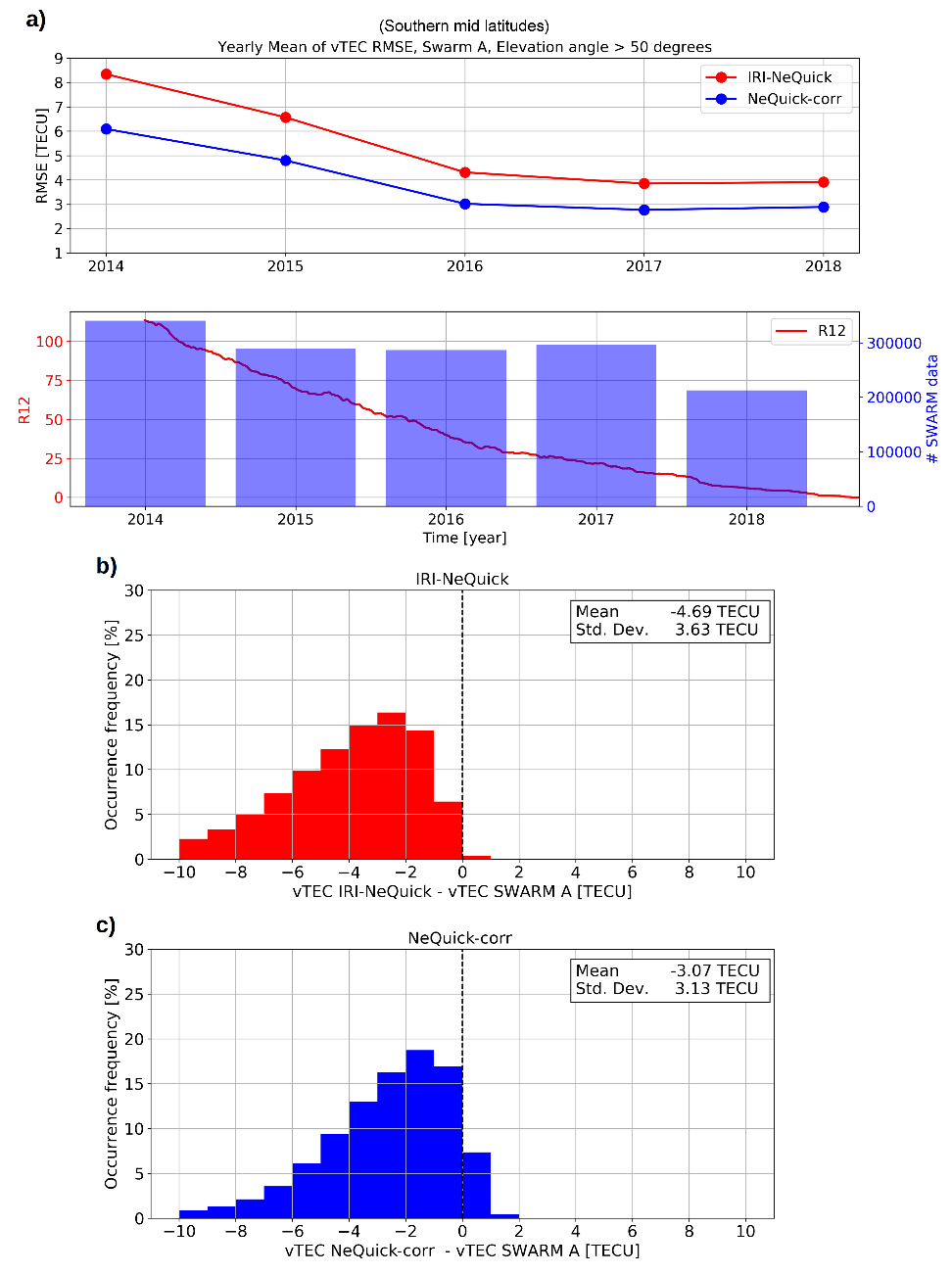


**Figure S4.** Same as Fig. 7 of the main paper but for Swarm A.


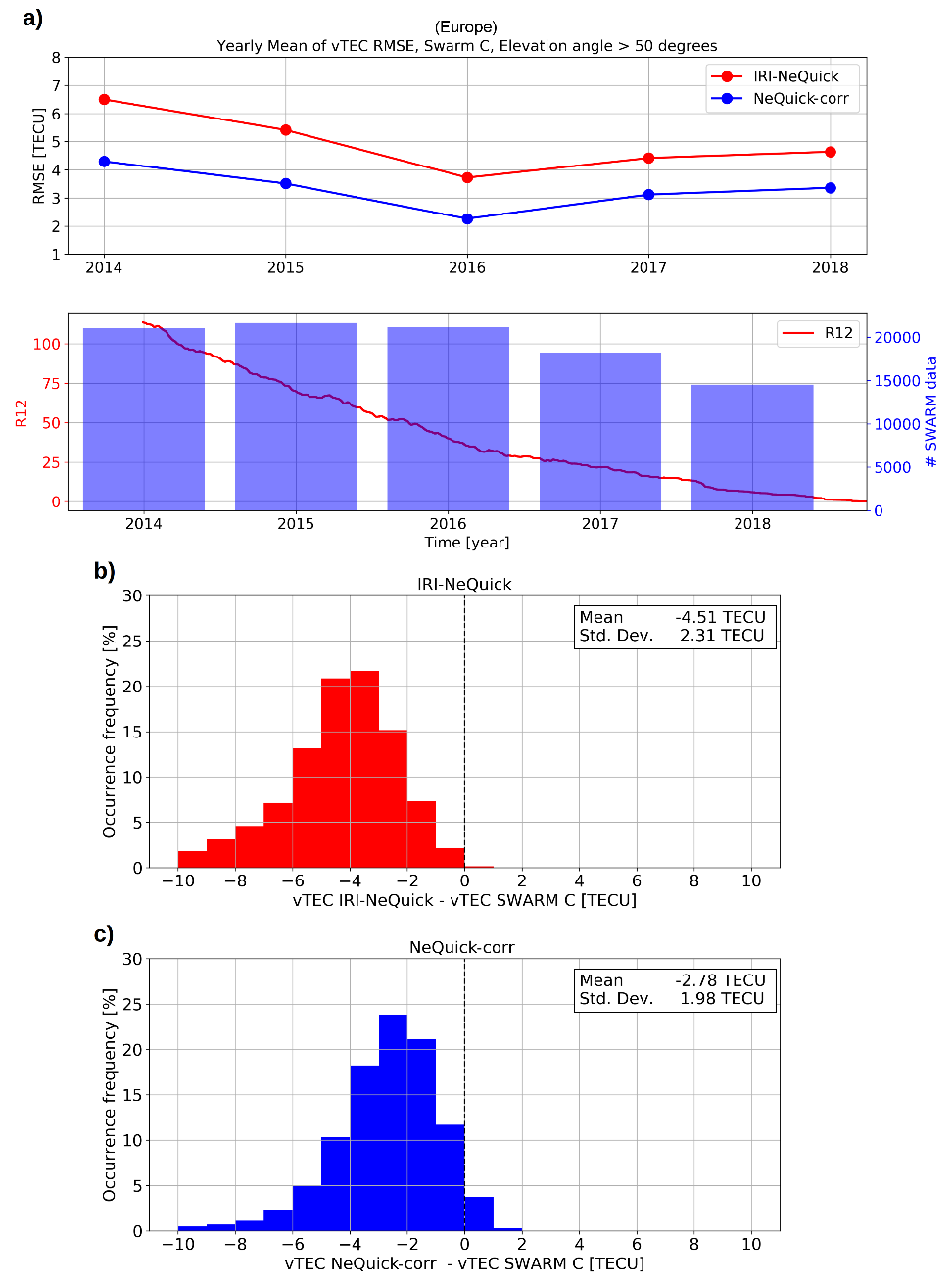


**Figure S5.** Same as Fig. 5 of the main paper but for Swarm C.


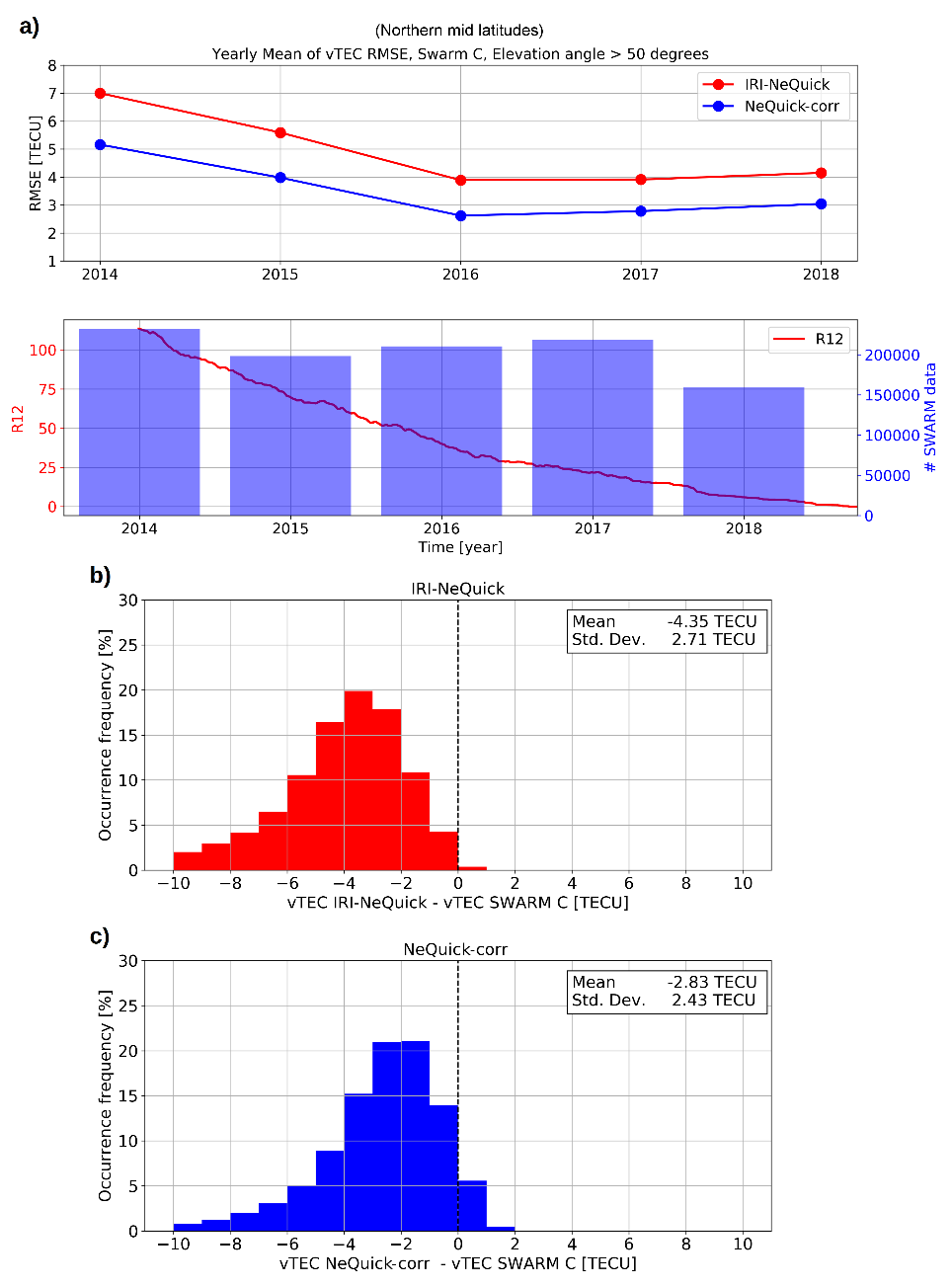


**Figure S6.** Same as Fig. 6 of the main paper but for Swarm C.


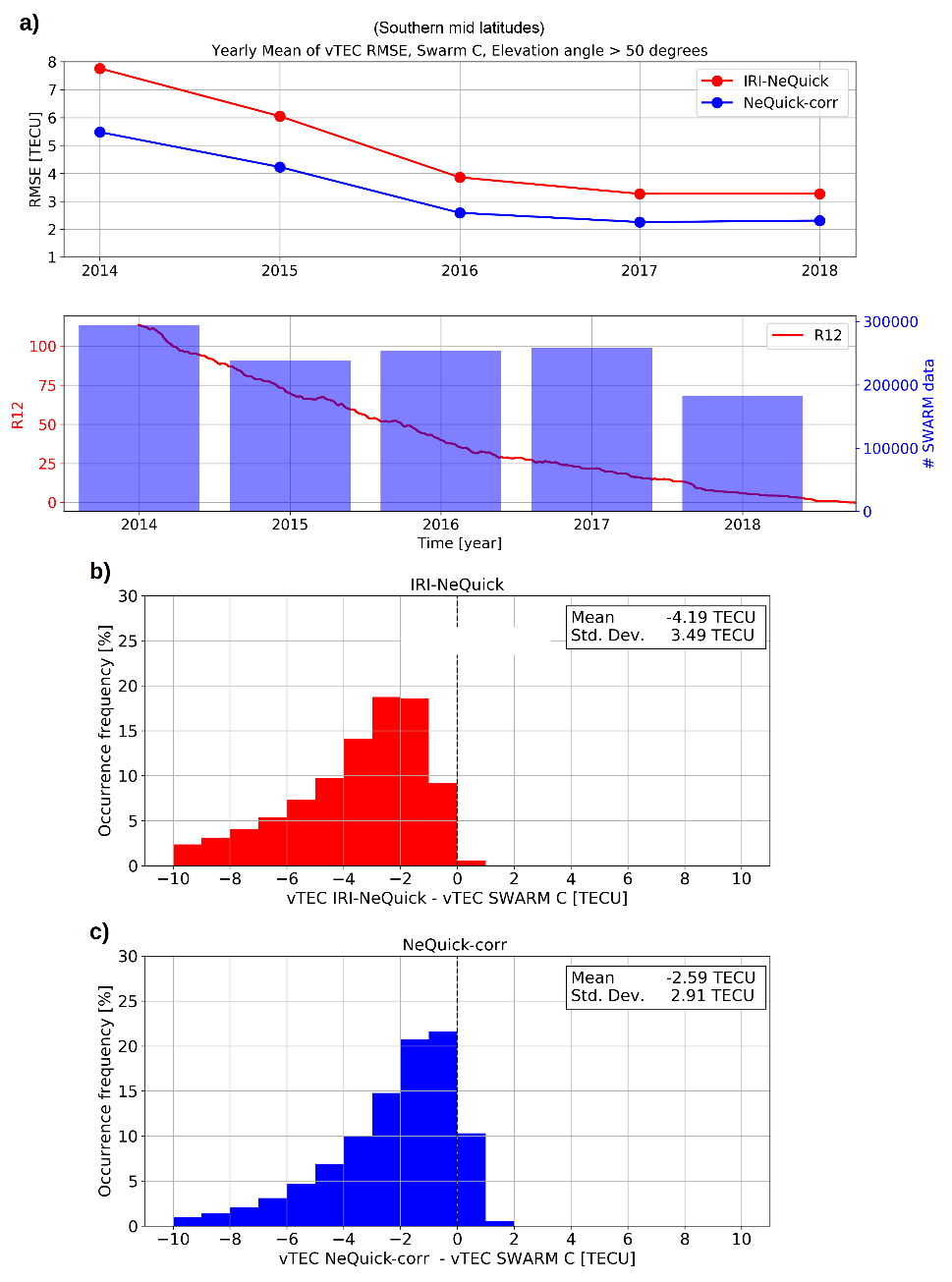


**Figure S7.** Same as Fig. 7 of the main paper but for Swarm C.
